# Haplotype Analysis of the First A4V-*SOD1* Spanish Family: Two Separate Founders or a Single Common Founder?

**DOI:** 10.3389/fgene.2019.01109

**Published:** 2019-11-08

**Authors:** Cecilia Garcia, Jose Manuel Vidal-Taboada, Enrique Syriani, Maria Salvado, Miguel Morales, Josep Gamez

**Affiliations:** ^1^ALS Unit, Neurology Department, Vall d’Hebron University Hospital, Barcelona, Spain; ^2^Vall d’Hebron Research Institute (VHIR), Barcelona, Spain; ^3^European Reference Network on Rare Neuromuscular Diseases (ERN EURO-NMD), Barcelona, Spain; ^4^Department of Medicine, Universitat Autònoma de Barcelona (UAB), Barcelona, Spain

**Keywords:** SOD1, A4V, p.A5V, amyotrophic lateral sclerosis, familial amyotrophic lateral sclerosis, ALS1, founder effect

## Abstract

Despite the genetic heterogeneity reported in familial amyotrophic lateral sclerosis (ALS) (fALS), Cu/Zn superoxide-dismutase (SOD1) gene mutations are the second most common cause of the disease, accounting for around 20% of all families (ALS1) and isolated sporadic cases (sALS). At least 186 different mutations in the SOD1 gene have been reported to date. The possibility of a single founder and separate founders have been investigated for D90A (p.D91A) and A4V (p.A5V), the most common mutations worldwide. High-throughput single nucleotide polymorphism genotyping studies have suggested two founders for A4V (one for the Amerindian population and another for the European population) although the possibility that the two populations are descended from a single ancient founder cannot be ruled out. We used 15 genetic variants spanning the human chromosome 21 from the SOD1 gene to the *SCAF4* gene, comparing them with the population reference panels, to demonstrate that the first A4V Spanish pedigree shared the genetic background reported in the European population.

## Introduction

About 10% of amyotrophic lateral sclerosis (ALS) cases are familial (fALS), and genetics is the discipline that has made the greatest contribution to understanding the complexity of the disease’s pathogenesis, with a major impact on clinical practice, especially in the field of genetic counseling. Since the discovery of mutations in the SOD1 gene linked to ALS in 1993, an increasing number of genes have been reported as associated with the disease, justifying the term “genetic heterogeneity of ALS”. At least 31 major genes and 2 different genetic loci with dominant, recessive, and X-linked patterns of inheritance have been identified for fALS, and an increasing number of susceptibility or modifying gene loci have been suggested for fALS and several sporadic ALS (sALS) cases. The ALSoD database currently lists genetic variants in 126 genes as associated to ALS. Gene–gene and gene–environment interactions have also been suggested as playing a major role in the disease’s appearance and phenotype (Andersen and Al-Chalabi, 2011; van Blitterswijk et al., 2012; Abel et al., 2013; Al-Chalabi et al., 2013; Leblond et al., 2014; Renton et al., 2014; Jones et al., 2015; Al-Chalabi et al., 2017; Brown and Al-Chalabi, 2017; Hardiman et al., 2017; van Es et al., 2017; Chia et al., 2018).

Mutations in the gene encoding Cu/Zn superoxide dismutase (SOD1) are the second most common cause of fALS cases worldwide, accounting for approximately 20% of all families. ALS1 is the designation for fALS linked to the SOD1 gene (MIM 105400). At least 186 different mutations in the SOD1 gene have been described, of which over 90% are missense, confirming its allelic heterogeneity (ALSoD^1^). The SOD1 mutations reported in ALS1 pedigrees are primarily associated with a dominant inheritance pattern and high penetrance, despite occasionally being found in apparently sporadic or recessive cases. Clinical heterogeneity, including gender predominance, age at symptom onset, site of onset, penetrance, and progression of the disease, has also been reported in many ALS1 families. The type of mutation and the resulting phenotype are strongly correlated in some cases (Andersen et al., 2003a; Andersen, 2003b; Andersen, 2006). However, information about the clinical–genetic correlations for most of these mutations is scarce. As a result, clinical and genetic data from families with ALS1 from around the world are being compiled in the constantly updated ALS database (ALSoD^1^). This database provides clinicians with information on research into the genetic characterization of ALS1 and other forms of familial ALS, as well as new candidate genes.

Epidemiological studies report that D90A is the most common mutation worldwide, although the most frequent mutation in North America is A4V (formally designated p.A5V, rs121912442), which accounts for 50% of North American ALS1 families (ALSoD^1^, Watanabe et al., 2000; Andersen, 2003b; Andersen, 2006). The most striking features of the p.A5V mutation are rapid progression, with a mean survival time of less than 2 years from clinical onset, predominant lower motor neuron symptoms, and its rarity in the European population. The possibility of two founder haplotypes—one Native American and another European—has recently been suggested (Rosen, 2004; Broom et al., 2008; Armon, 2009; Saeed et al., 2009; Tang et al., 2018). The p.A5V*-SOD1* mutation has been described in a very small number of families in Europe, and never in the Spanish population.

Here we report the clinical characterization and high-throughput single nucleotide polymorphism (SNP) genotyping of the first p.A5V (A4V) Spanish ALS kindred with high penetrance, predominant lower motor neuron involvement, fast progression, short survival times, and no cognitive impairment.

## Material and Methods

### Subjects

This study was carried out following the protocol approved by the Hospital Universitari Vall d’Hebron Institutional Review Board with written informed consent from all subjects in accordance with the Declaration of Helsinki.

**Figure 1:**
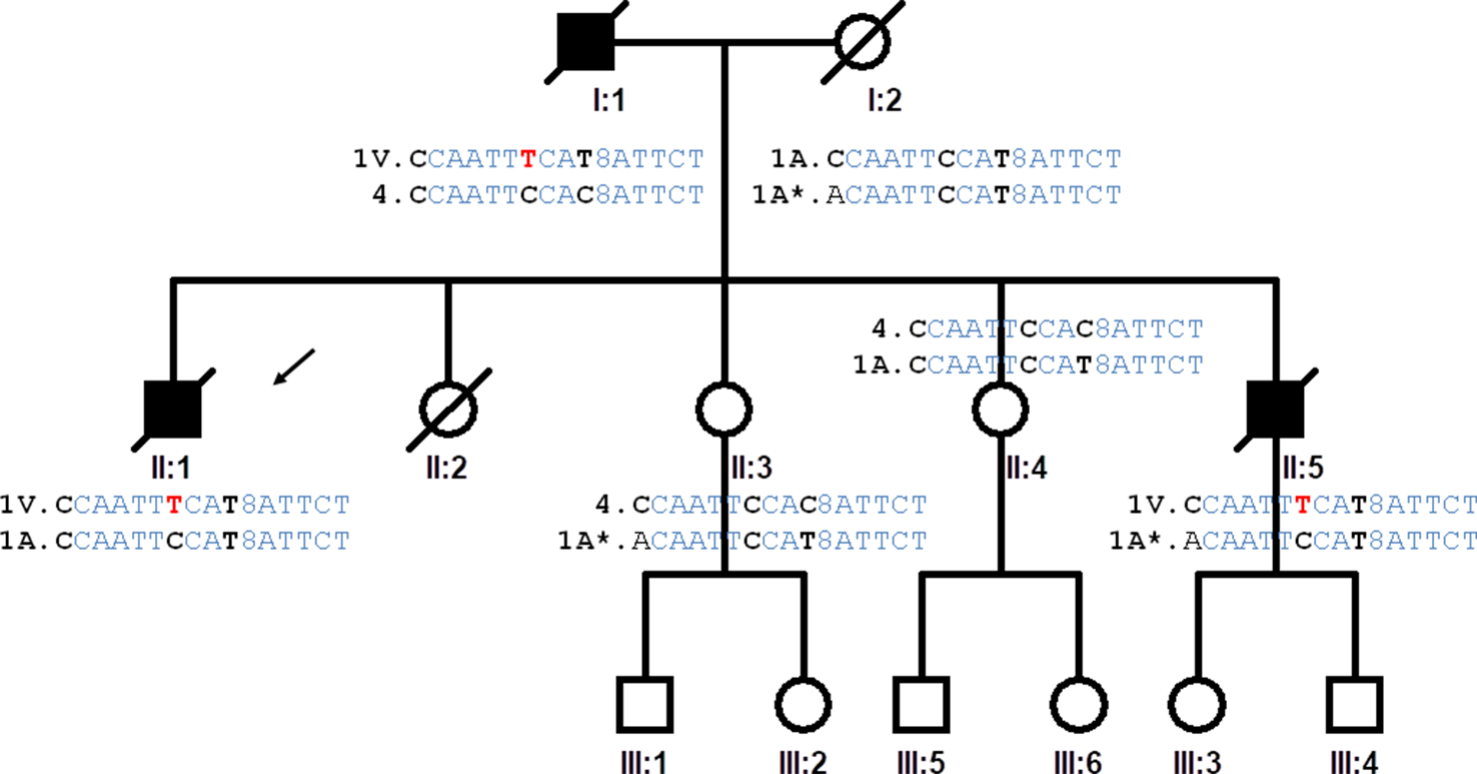
Pedigree of the family studied. The proband is indicated by an arrow (II:1). The haplotypes of the progenitors are inferred from the haplotypes of the offspring. Highlighted in red the genetic variation causing A5V mutation, in bold the single nucleotide polymorphisms that determine the differences between haplotypes of the IBS population. Haplotype (variation order): rs4817415, rs2070422, rs1008270, rs9974610, rs2173962, rs202445, rs121912442, rs4816405, Rs2070424, rs1041740, CA_repeat, rs2833475, rs16988427, rs2833481, rs2070423, rs2833483.

Our pedigree originated in north-western Spain. The simplified pedigree of the family is shown in **Figure 1**. We examined two ALS patients and two healthy individuals in the pedigree after obtaining informed consent. There were no skipped generations. The affected individuals were clinically characterized according to gender, age at onset, initial topography, signs of dementia, and survival time (**Supplementary Table S1**).

The proband, a 53-year-old male (II:1), noticed weakness in his left leg beginning 7 months previously, which he attributed to a traumatism he had experienced when working in construction. An initial electromyographic study showed denervation in all four extremities. Transcranial magnetic stimulation and brain and spinal MRI were normal. Cerebrospinal fluid, hemogram, biochemical screening, and serological tests for neurotropic infectious diseases all presented normal values, except for *creatine kinase*, which was 735 U/l (normal values < 135). The patient subsequently experienced pain, with frequent falls in the months after his first examination, when muscle strength in his upper limbs and right lower limb was completely normal. His Amyotrophic Lateral Sclerosis Functional Rating (ALSFRS-R) score was 44. In the next visit, 3 months later, we observed weakness and amyotrophy in the left upper limb, particularly in the proximal muscles. We observed generalized fasciculations, which were more marked in the muscles on the left side of the body, where it was difficult to elicit deep tendon reflexes. Eight months after the initial visit, the patient was admitted to the emergency ward complaining of dyspnea and orthopnea, and non-invasive mechanical ventilation was fitted. The patient was offered a gastrostomy, but declined. The patient died 19 months after symptom onset.

Two years later, the proband’s 47-year-old brother (II:5) came to our clinic due to presenting clumsiness and difficulties climbing stairs, running and walking. He had experienced recurring episodes of cramp in both calves in the 2 months before his visit. We observed weakness and amyotrophy in the proximal muscles of the right lower limb and a minimal loss of muscle mass in the first interosseous of the right hand. Deep tendon reflexes were normal, except for the patellar and Achilles reflexes. Fasciculation was observed in the musculature of the four extremities. His ALSFRS-R rating at this point was 39. Like his brother, neurophysiological studies showed signs of denervation in three regions and an absence of upper motor neuron signs. The patient declined any follow-up, life support, ventilation or nutritional measures. He died 17 months after clinical onset.

In their family, their two sisters requested genetic screening for familial forms of ALS, as they remembered that their father had died at 57 years of age due to respiratory insufficiency after 2 years of weakness in the upper limbs. To the best of our knowledge, this pedigree contains no other cases of neurodegenerative disorders, including frontotemporal dementia and Parkinson’s disease.

### Genetic Studies

#### Selection of Single Nucleotide Polymorphisms for Analysis of the *SOD1* Haplotype

In order to saturate the *SOD1* region with genetic markers, we performed a scientific literature search of haplotypes previously described in the *SOD1* gene. We consulted various databases (1K Genomes, dbSNP, and Ensembl Web sites) for SNPs in the *SOD1* region of the IBerian populations in Spain (IBS) population, and analyzed 14 SNPs and 1 repeat. Thirteen SNPs were selected from the results of 79 A4V families (Broom et al., 2008) (rs4817415, rs2070422, rs1008270, rs9974610, rs2173962, rs4816405, rs2070424, rs1041740, rs2833475, rs16988427, rs2833481, rs2070423, rs2833483); and 1 additional SNP (rs202445) and a CA repeat from a study of 54 patients (Saeed et al., 2009) and genetic data from an isolated Chinese case (Tang et al., 2018). The locations of all markers and their previous designations are shown in **Supplementary Table S2**.

#### Genotyping of Normal and Mutant p.A5V Alleles in SOD1

The SNP rs121912442 (*SOD1* p.A5V) region was amplified by PCR and the allele detected by Sanger sequencing. DreamTaq Master Mix was used under standard conditions according to the manufacturer’s instructions. Primer sequences and PCR cycling conditions are shown in **Supplementary Table S3**. PCR products were purified with ExoSAP-IT™ (Thermo Fisher Scientific Inc.) prior to automated Sanger sequencing using a BigDye^®^ v3.1 terminator Cycle Sequencing Kit in an ABI3730XL^®^ (Thermo Fisher Scientific Inc., USA). DNA sequences were analyzed using the Finch TV 1.5.0 software.

#### Genotyping of Single Nucleotide Polymorphisms and Microsatellite

PCR Amplification of CA-repeat region was performed, and the products were analyzed by Sanger sequencing. The results were validated by Fragment Size Analysis using an ABI3730XL^®^ system and GeneMapper 4.0 Software (Applied Biosystems). Most SNPs were genotyped by PCR amplification and Sanger sequencing as indicated above. In other cases, SNPs were detected by allele-specific PCR using standard conditions and an internal PCR control. Their primers and annealing temperature are shown in **Supplementary Table S3**. PCR primers were designed using Primer3 version 4.0. (Untergasser et al., 2012).

The entire coding regions of SOD1, FUS, TARDBP, and PFN1 genes and the *C9orf72* expansion were screened using Sanger sequencing.

#### Analysis of Data

LDhap software (Machiela and Chanock, 2015) was used with a 1K Genomes dataset to identify inferred haplotype blocks in the Iberian (IBS) population. Linkage disequilibrium between SNPs was calculated using LDMatrix software (LDLink). The *SOD1* p.A5V haplotypes detected in our Spanish patients were compared with the haplotypes associated in American, Swedish, and Chinese populations (Broom et al., 2008; Tang et al., 2018). The genetic data for the comparisons with the African (ACB, ASW, ESN, GWC, LWK, MSL, YRI), Asian (CHB, CHS, CDX, JPT, KHV), European (CEU, FIN, GBR, IBS, TSI), and Mixed American (CLM, MXL, PEL, PUR) populations were obtained from all the available subjects (n = 2,312 individuals from 19 populations, including 102 IBS subjects) of the 1K Genomes Project (phase 3, version 5) (The 1000 Genomes Project Consortium, 2015). Haplotype assembly was carried out by manual phasing of the alleles from the different variants analyzed.

## Results

### Clinical Characteristics of the p.A5V-*SOD1* Pedigree

The clinical phenotype in the affected members in our family—a mean age of onset of 51.7 years, spinal onset, predominant lower motor neuron signs, and a survival time of 20 months—is consistent with the phenotype in the American, Italian, and Swedish families. No cognitive impairment in either brother was detected in the neuropsychological test (**Supplementary Table S1**).

### Genetic Results

Mutation analysis of the *SOD1* gene by direct PCR sequencing revealed a C-to-T transition at nucleotide position 14 (c.14C > T, **Supplementary Figure S1**) leading to a p.A5V (rs121912442, A4V in the old nomenclature) sequence change at protein level in the two affected ALS patients: the proband (individual II:1) and his brother (individual II:5) (**Table 1**). This exon 1 mutation was not found in the two healthy members of the family (individuals II:3 and II:4) who requested details of their genetic situation (**Supplementary Figure S1**).

**Table 1:** SOD1 p.A5V mutation founder haplotype in IBS population compared to other populations.

RS number	SOD1 A5V founder haplotype				Patients genotype	
	USA*	SWE*	CHN**	IBS	II:1	II:5
**rs4817415**	C	C	−	C	C/C	C/A
**rs2070422**	T	C	C	C	C/C	C/C
**rs1008270**	A	A	−	A	A/A	A/A
**rs9974610**	A	A	−	A	A/A	A/A
**rs2173962**	T	T	−	T	T/T	T/T
**rs202445**	-	-	−	T	T/T	T/T
**rs121912442**	T	T	T	T	T/C	T/C
**rs4816405**	G	C	C/G	C	C/C	C/C
**rs2070424**	G	A	A	A	A/A	A/A
**rs1041740**	C	T	C/T	T	T/T	T/T
**rs2833475**	G	A	A	A	A/A	A/A
**rs16988427**	C	T	T/C	T	T/T	T/T
**rs2833481**	C	T	T	T	T/T	T/T
**rs2070423**	T	C	C/T	C	C/C	C/C
**rs2833483**	C	T	T/C	T	T/T	T/T

*Data from Broom et al., 2008. **Data from Tang et al., 2018. (-) Not available.

No mutations were detected in the *FUS*, *TARDBP*, and *PFN1* genes, and no *C9orf72* expansions were identified in the two ALS patients.

### Haplotype Analysis

We analyzed the 15 genetic variants spanning the human chromosome 21 from the *SOD1* gene to the *SCAF4* gene (**Supplementary Table S2**). The genetic markers around the *SOD1* gene were genotyped to infer the *SOD1* haplotypes of the ALS patients and the family members. Based on the genotypes of the four siblings, it was possible to identify four different inferred haplotypes present in the family and propose the haplotypes of the affected father and the mother (**Figure 1**).

**Figure 2:**
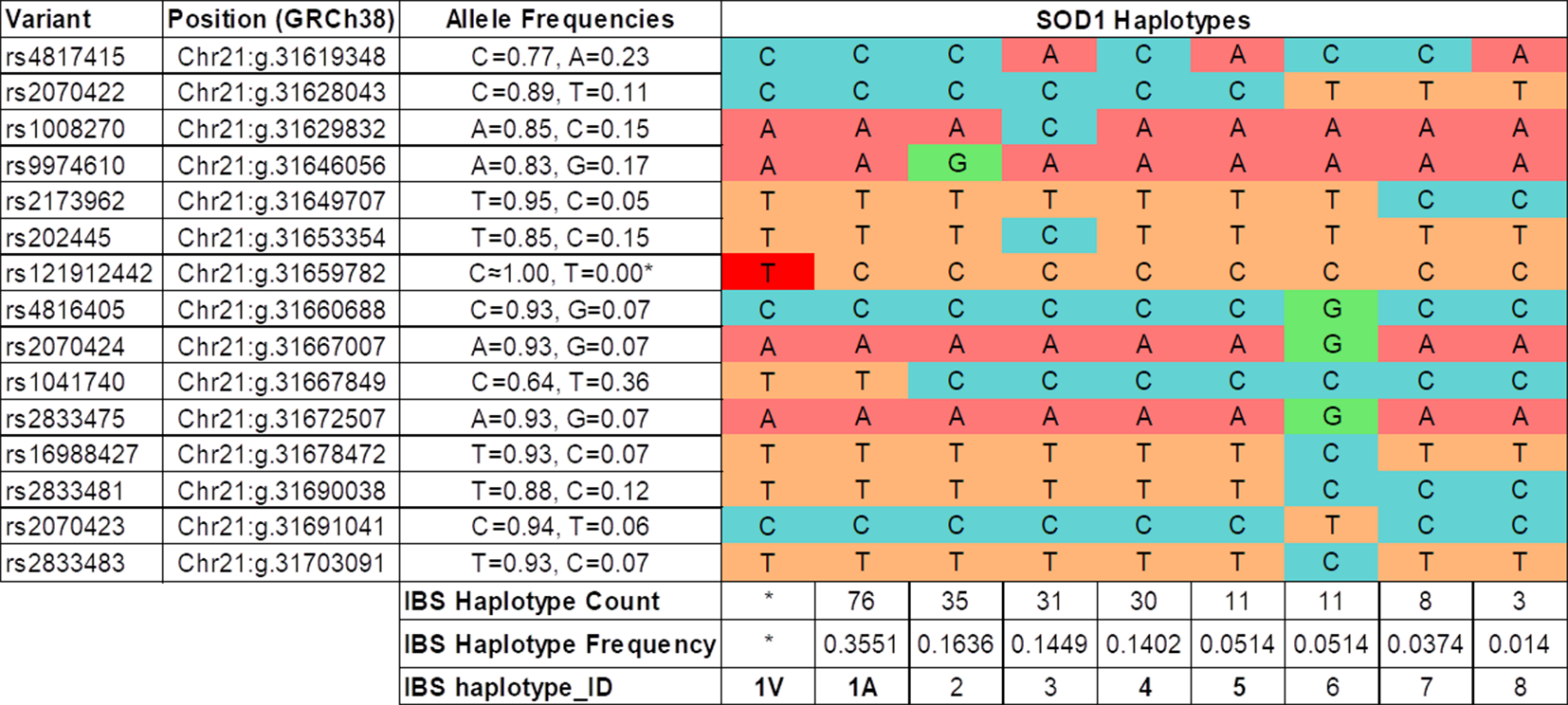
Haplotypes inferred in the IBS population (1K Genomes Project). The IBS haplotype_ID codification is based on the frequency of the haplotype in the IBS population, and in the case of the most frequent (IBS haplotype_ID = 1) the presence of the *SOD1* p.A5V protein mutation (1V). (*) The haplotype 1V and allele rs121912442T are only present in the Spanish ALS patients with the *SOD1* p.A5V mutation.

Fourteen of the 16 SNPs analyzed in the *SOD1* gene were detected in subjects of the IBS population from the 1K Genomes Project. Eight phased haplotypes of the *SOD1* region were inferred in the IBS population with different frequencies (**Figure 2**). The haplotypes were named (1 to 8) according to their frequency in the population, ranging from higher to lower frequency (**Figure 2**). The mutation p.A5V-*SOD1* detected in the ALS patients is only compatible with haplotype 1 (haplo1V), which is the most common in the IBS population (haplo1A) (**Figure 2** and **Table 1**). According to the nomenclature proposed, the family has the IBS haplotypes 1A, 1V, 4, and 1A* (**Figure 1**).

The linkage disequilibrium of these 14 SNPs was calculated for the IBS population (**Supplementary Figure S2**). A conserved haplotype block, in high linkage disequilibrium, was detected between the start of intron 1 and the end of *SOD1*, and including the *SCAF4* gene. The *SOD1* exon 1, including the c.14C > T (p.A5V) mutation, is located outside this conserved haplotype in the IBS population (**Supplementary Figure S2**). Using data for all the populations deposited in the 1K Genomes Project, the analysis of linkage disequilibrium showed the same results for the conservation of this *SOD1* haplotype block (data not shown).

The founder p.A5V-*SOD1* haplotype in our patients is the same as the one found in the Swedish population, and differs from the founder haplotype observed in patients from North America (**Table 1**).

**Figure 3:**
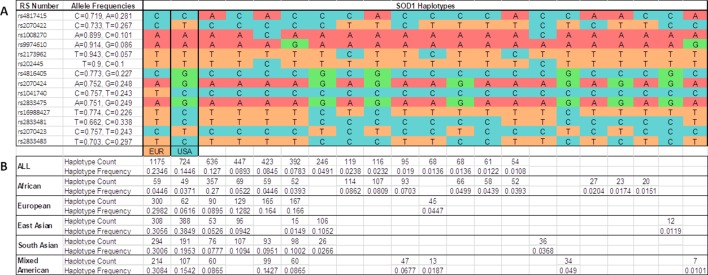
Inferred haplotypes around the *SOD1* gene detected in all populations from the 1K Genomes Project. **(A)** 21 inferred haplotypes. **(B)** Haplotype frequencies and counts are shown for each subpopulations: African (ACB, ASW, ESN, GWC, LWK, MSL, YRI); European (CEU, FIN, GBR, IBS, TSI); East Asian (CHB, CHS, CDX, JPT, KHV); South Asian (GIH, PJL, BEB, STU, ITU); Mixed American (CLM, MXL, PEL, PUR); all (African + European + East Asian + South Asian + Mixed Americans). EUR, haplotype base for *p.A5V*-EU. USA, haplotype base for *p.A5V*-USA.

We determined all the inferred *SOD1* haplotypes for these 14 SNPs in the IBS, European, Asian, African, and Mixed American populations, using all the available subjects from the 1K Genomes Project as a control group (**Figure 3**). A total of 21 phased inferred haplotypes were detected in all the populations. The European haplotype (p.A5V-EUR) was the most common in all the populations. The American haplotype (p.A5V-USA) was the second most common worldwide. However, both haplotypes appear with similar frequencies in the Asian population (0.386 for p.A5V-EUR, 0.385 for p.A5V-USA) (**Figure 3**).

## Discussion

### Redefining the p.A5V-SOD1 Phenotype. Reasons for the Disease’s Aggressive Course

Over 186 *SOD1* mutations have been reported in amyotrophic lateral sclerosis (ALSoD). Previous epidemiological studies suggest that p.D91V is the most common worldwide, followed by p.I113T and p.A5V. The latter is the most commonly reported in the United States, accounting for 41% of the mutations identified in that population. It is one of the most aggressive mutations, as the mean survival time is under 2 years. The mutation has rarely been described among European populations. This is the ﬁrst time that the p.A5V-*SOD1* has been reported in the Spanish population.

With the rare exception of a 73-year-old male presenting facial diplegia and unilateral vocal cord paralysis as the initial symptoms of ALS (Salameh et al., 2009), the phenotype associated with p.A5V in the American population is characterized by rapid progression and short survival. Patients present predominantly lower motor neuron signs, with limb weakness in 51% of patients, and bulbar symptoms in less than 11% of cases (Cudkowicz et al., 1997; Juneja et al., 1997; Andersen, 2003b; Andersen, 2006; Broom et al., 2008; Saeed et al., 2009). In a recent review of 57 p.A5V-*SOD1* cases, the mean survival time was 1.4 years, with clinical onset at a mean age of 50.0 years, and a male-female ratio of 1.3:1. Other characteristics associated with this *SOD1* mutation include fast progression, predominantly spinal onset, high penetrance, involvement of primarily lower motor neuron signs, and an absence of dementia (Bali et al., 2017).

However, the cases reported in European families differ slightly from those in America. For example, Andersen reported the clinical characteristics of a Swedish family (Andersen et al., 1997; Andersen, 2007). One case had leg onset, another had hand onset, another had shoulder onset, and a further case had bulbar onset. These individuals were aged between 56 and 68 years old, and survival ranged from 8 months to 2 years. In the members of the two Italian families reported in 2001, information was available for two probands. One had shoulder onset at 55 years old, and died 8 months later. The other proband presented hand onset at 57 years old. No information about this individual’s survival time is available, although the course was rapidly progressive (Gellera et al., 2001). Our family’s phenotype—a mean age of onset of 51.7 years, spinal onset, predominant lower motor neuron signs, no cognitive alteration, and a survival time of 20 months—is consistent with the phenotype in these European families. Interestingly, none of the women inherited the mutation. Whether this was due to chance or a protective factor is unknown.

The reasons why p.A5V-*SOD1* predicts fast progression and short survival are unknown. *In vitro* studies have pointed to this mutation leading to a decline in dimerization capacity, a loss of metalation (a 30-fold decrease in zinc-binding affinity), and aberrant oligomerization, leading to misfolding and aggregation in the form of insoluble toxic inclusions within motor system cells, a key pathological hallmark of ALS. There is some variability in the propensity of *SOD1* mutants to aggregate, which could be related to the duration of the disease. High-aggregation propensities have been described for p.A5V, which could be the factor responsible for the disease’s short duration (Prudencio et al., 2009; Zhao et al., 2014; Farrawell et al., 2018; Maurel et al., 2018; Srinivasan and Rajasekaran, 2018).

Another alternative and complementary hypothesis to explain the aggressive nature of p.A5V is its position in the tertiary structure of the protein. Interestingly, of the 30 mutations reported in exon 1, most of those in residue A5 (A4 in the previous nomenclature) predispose to a rapid progression and survival of under 24 months (Syriani et al., 2009). According to the tertiary structure of *SOD1*, residue A5 is located in the first β-strand that forms part of the dimer interface. A5 mutations affect *SOD1* dimerization and/or destabilize the Greek key β-barrel because A5 packs into the *SOD1* monomer’s hydrophobic core (Getzoff et al., 1990; Deng et al., 1993; Cardoso et al., 2002; Galaleldeen et al., 2009; Schmidlin et al., 2009; Schmidlin et al., 2013; Kumar et al., 2018).

### Founder Effect

Two earlier studies investigated a possible founder effect in the cases of North American and European origin. Following these haplotype studies, there is evidence to suggest at least two different origins: one European (probably a Scandinavian founder effect) and one Amerindian (Native Americans). The latter presents in 82% of North American families with the p.A5V-*SOD1* mutation. In European populations, p.A5V-*SOD1* has been reported in Scandinavia (three cases) and Italy (six cases). An isolated case has recently been reported in China, which shares most of the European haplotype. These findings could be interpreted in terms of the existence of various founders for p.A5V patients worldwide, rather than one founder, as previously assumed (Broom et al., 2008; Saeed et al., 2009). Some authors suggest that the European founder effect is older than the Amerindian, hypothesizing that the American families were in fact descendants of the European founder. Other authors argue that the European origin is different from the American one (Rosen, 2004; Armon, 2009).

After analyzing the *SOD1* haplotypes in different populations (European, Asian, African, and all the populations analyzed in the 1K Genomes Project), we found that the *SOD1* haplotype in which the p.A5V mutation is located in European fALS patients (p.A5V-EU) is the most common in the population worldwide, and the haplotype for American p.A5V-*SOD1* patients (p.A5V-USA) is the second most common. These variants appear to be linked to the most common haplotypes, and not linked to rare haplotypes. The haplotype p.A5V-USA is the most common (0.385) in the East Asian population and the p.A5V-EU haplotype is the second most common (0.306), with little difference between their allelic frequencies. However, in the European population, the haplotype p.A5V-EU is the most common haplotype (0.298), far below the frequency of the haplotype p.A5V-USA (0.062). The haplotypes p.A5V-EU (0.037) and p.A5V-USA (0.045) are rare in the African populations (as shown in **Figure 3**).

Based on these data, we propose two possible hypotheses for the origin of this mutation in two different haplotypes:

#### A Single Origin for All p.A5V*-SOD1* Families Worldwide

Information from previous linkage analyses suggests an origin for p.A5V around 13,000 years ago, probably in Asia. The p.A5V mutation appears in the p.A5V-EU haplotype and would have disseminated in Europe and Asia. A homologous recombination could have occurred in an ALS American family, leading to the linkage of the p.A5V mutation to the p.A5V-USA haplotype. A founder effect in North America could explain the p.A5V mutation associated to the p.A5V-USA haplotype in most (82%) cases in the American families (Rosen et al., 1994; Rosen, 2004; Broom et al., 2008; Armon, 2009; Saeed et al., 2009) and to the p.A5V-EU in only 18% of the other fALS cases. Only the p.A5V mutation linked to the haplotype p.A5V-EU is detected in Asia and Europe. Interestingly, linkage analysis of the haplotype of *SOD1* SNPs, a highly conserved haplotypic block (with a strong correlation) is observed after exon 1, but does not contain exon 1.

#### Several Origins (One for Asians, One for Americans, and Perhaps One or More for Europeans)

Previous studies investigating the haplotypic region around *SOD1* have suggested different mutational events to explain the mutation in USA and European ALS patients (Rosen, 2004; Broom et al., 2008, Eisen et al., 2008; Saeed et al., 2009). It is possible that the mutation in A5 was generated in the haplotypes p.A5V-USA and p.A5V-EU independently, since the two haplotypes are the most frequent in the population worldwide, and the locus at codon 5 of *SOD1* would be prone to mutations (a hotspot) since four additional mutations (p.A5T, p.A5S, p.A5F, and p.A5P) have been described in this codon (Syriani et al., 2009; Pratt, 2014).

From the molecular point of view, the most parsimonious hypothesis would be a single origin for the A5V mutation. Our hypothesis of a common founder for p.A5V is supported by research investigating who first colonized America and when they did so. These genetic studies showed that the first inhabitants of the Americas came from a single Siberian population, who used the Bering Land Bridge to migrate from Beringia to the Americas sometime after 16,500 years ago (Bonatto and Salzano, 1997; Goebel et al., 2008). We propose a Eurasian origin for the A5V mutation in the haplotype p.A5V-EU. A Eurasian individual could have introduced the A5V mutation of the haplotype p.A5V-EU in America, and the mutation could have recombined in their descendants to obtain the p.A5V-USA haplotype linked to the p.A5V mutation. A genetic drift in America could therefore explain the founder effect of p.A5V in the haplotype p.A5V-USA being the most frequent ALS1 mutation in USA. A similar genetic phenomenon was described for the D90A mutation. Initial studies proposed two different founder effects (one for heterozygous cases and one for homozygous cases) (Al-Chalabi et al., 1998). After increasing the sample set and the markers, they concluded that there was a single founder for all cases. These studies for this mutation showed that the D90A mutation arose in Eurasia approximately 20,000 years ago (Parton et al., 2002).

Asian or Amerindian origins have been proposed for the A5V mutation based on their high frequency in USA and the haplotype p.A5V-USA being the most frequent haplotype in Asian populations (Rosen 2004; Broom et al., 2008; Saeed et al., 2009). The hypothesis of these two origins (Amerindian and Asian) is contradicted by the failure to detect the A5V mutation in the p.A5V-USA haplotype in ALS cases from Africa, Europe, Asia, and among Native Americans. Current data, including our results, indicate that the A5V mutation cases have been associated to the p.A5V-EU haplotype in different populations from Europe, USA, and Asia.

The *SOD1* haplotypes inferred from the IBS population enabled us to identify the haplotypes of the four members of a Spanish ALS family of Caucasian origin with two affected brothers with the p.A5V-*SOD1* mutation. The *SOD1* haplotype associated to the A5V mutation in our Spanish cases is the most frequent in the IBS and European populations. To our knowledge, our Spanish family is unrelated to the other p.A5V reported cases from Sweden (three subjects from one family) or Italy (six subjects from two families). A more detailed haplotype analysis of the European fALS cases with this mutation will be necessary to investigate whether there is a common origin for the p.A5V-*SOD1* mutation in the European ALS cases, or if it is a consequence of different mutational events. This research could be carried out through an international collaborative consortium to enroll and analyze these ALS families, as in the study suggested by an Italian group (Gellera et al., 2001).

In conclusion, this is the ﬁrst report on the p.A5V-*SOD1* mutation in the Spanish population. The age at onset, site of onset, and survival were similar to those reported mainly in North American kindreds, in a few European families and in one Asian individual. SNP and haplotype analyses identify 21 haplotypes worldwide for the *SOD1* genomic region. Our family shares the haplotype reported in the founder European effect rather than the more frequent Amerindian haplotype.

## Data Availability Statement

Publicly available datasets were analyzed in this study. This data can be found here: http://www.internationalgenome.org/data.

## Ethics Statement

This study was carried out in accordance with the recommendations of the Hospital Universitari Vall d’Hebron Institutional Review Board (VdHIRB) with written informed consent from all subjects. All subjects gave written informed consent in accordance with the Declaration of Helsinki. The protocol was approved by the VdHIRB.

## Author Contributions

JG and JV-T conceived the study. JG and MS collected the clinical information. CG, JV-T, ES, MM, and JG analyzed the data. JV-T and CG conducted the bioinformatics analysis. CG, JV-T, and JG drafted the manuscript. CG and JV-T contributed equally to this work. All the authors approved the final version of manuscript.

## Funding

This study has been supported by Instituto de Salud Carlos III (grant numbers PIS-FEDER PI16/01673 and PI19/00593). JG and JV-T are the recipients of grant 2017SGR00939 from Agència de Gestió d’Ajuts Universitaris i de Recerca (AGAUR) of the Generalitat de Catalunya.

## Conflict of Interest

The authors declare that the research was conducted in the absence of any commercial or financial relationships that could be construed as a potential conflict of interest.

## Footnotes

1 http://alsod.iop.kcl.ac.uk/mutations/mutationsFoundGeneOnly.aspx?gene_id=SOD1. Web site access on Oct 8th 2019.
