# Supplementary material for: Haplotype Analysis of the First A4V-SOD1 Spanish Family: Two Separate Founders or a Single Common Founder?
Source: Front Genet. 2019 Nov 8;10:1109. doi: 10.3389/fgene.2019.01109 (PMC6857184; doi:10.3389/fgene.2019.01109)

Supplementary Figure S1. Electropherograms of the family members analyzed, showing the SOD1 c.14C>T mutation (p.A5V, A4V, rs121912442)

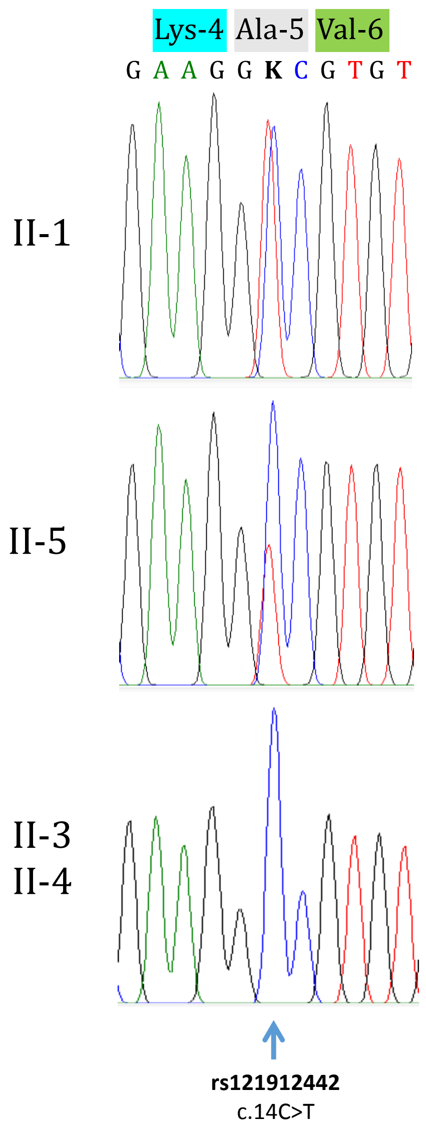

Supplement: Supplementary file 1 [file Image_1.pdf]
