# Supplementary material for: Haplotype Analysis of the First A4V-SOD1 Spanish Family: Two Separate Founders or a Single Common Founder?
Source: Front Genet. 2019 Nov 8;10:1109. doi: 10.3389/fgene.2019.01109 (PMC6857184; doi:10.3389/fgene.2019.01109)

**Supplementary Figure S2.** Linkage disequilibrium analysis showing the haplotype blocks observed in the SOD1-SCAF4 genomic region of the IBS population using LDlink software. a) Linkage disequilibrium correlations observed between the SNPs analyzed. b) Location of the SNPs analyzed in the human chromosome 21 (coordinates are referred to human genome GRCh37 version). Location of the A5V mutation (*) and the CA_Repeat variant (#).

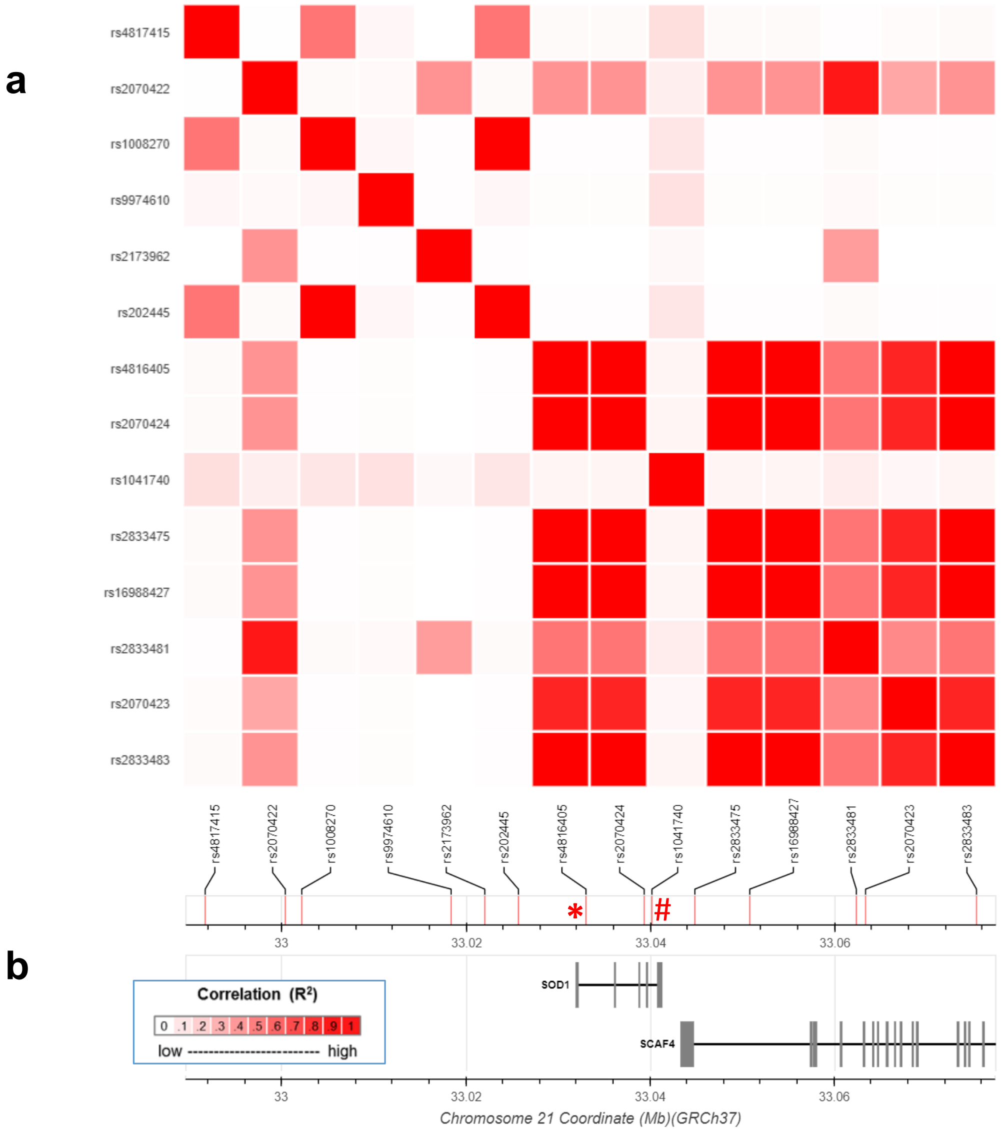

Supplement: Supplementary file 2 [file Image_2.pdf]
