# Supplementary material for: Haplotype Analysis of the First A4V-SOD1 Spanish Family: Two Separate Founders or a Single Common Founder?
Source: Front Genet. 2019 Nov 8;10:1109. doi: 10.3389/fgene.2019.01109 (PMC6857184; doi:10.3389/fgene.2019.01109)
Supplement: Supplementary file 3 [file Table_1.pdf]

Supplementary Table S1. Clinical data of p.A5V Spanish family members.

individual	Gender (M/F)	genotype	Symptoms	Site of onset	Phenotype	Cognitive impairment	Age at onset (years)	Survival (months)
I.1	M	p.A5V/WT	Yes	LLL	Predominant LMN syndrome	No	55	23
I.2	F	UNK	No	No	NA	NA	No	NA
II.1	M	p.A5V/WT	Yes	LLL	Predominant LMN syndrome	No	53	19
II.2	F	WT/WT	No	No	NA	NA	No	NA
II.3	F	WT/WT	No	No	NA	NA	No	Alive
II.4	F	WT/WT	No	No	NA	NA	No	Alive
II.5	M	p.A5V /WT	Yes	RLL	Predominant LMN syndrome	No	47	17

Abbreviations: F= female; LLL= left lower limb; M= male; NA= not applicable; RLL= right lower limb, UNK= unknown.
