# Supplementary material for: Haplotype Analysis of the First A4V-SOD1 Spanish Family: Two Separate Founders or a Single Common Founder?
Source: Front Genet. 2019 Nov 8;10:1109. doi: 10.3389/fgene.2019.01109 (PMC6857184; doi:10.3389/fgene.2019.01109)
Supplement: Supplementary file 4 [file Table_2.pdf]

Supplementary Table S2. Correspondences between rs numbers, genomic positions (GRCh37 and GRCh378) and the Alias_ID used in previous papers by Broom et al. (2008), Saeed et al. 2009 and Tang et al. (2018).

Alias_ID	rs number	Position (GRCh38)	Position (GRCh37)
SNP2	rs4817415	Chr21:31619348	Chr21:32991661
SNP3	rs2070422	Chr21:31628043	Chr21:33000356
SNP17	rs1008270	Chr21:31629832	Chr21:33002145
SNP4	rs9974610	Chr21:31646056	Chr21:33018369
SNP20	rs2173962	Chr21:31649707	Chr21:33022020
Frg2 (*)	rs202445	Chr21:31653354	Chr21:33025667
A4V	rs121912442	Chr21:31659782	Chr21:33032095
SNP33	rs4816405	Chr21:31660688	Chr21:33033001
SNP12	rs2070424	Chr21:31667007	Chr21:33039320
SNP6	rs1041740	Chr21:31667849	Chr21:33040162
CA_repeat (*)	NA	Chr21:31670634-31670651	Chr21:33042947-33042964
SNP13	rs2833475	Chr21:31672507	Chr21:33044820
SNP14	rs16988427	Chr21:31678472	Chr21:33050785
SNP9	rs2833481	Chr21:31690038	Chr21:33062351
SNP16	rs2070423	Chr21:31691041	Chr21:33063354
SNP10	rs2833483	Chr21:31703091	Chr21:33075404

(*) Polymorphisms from Saeed et al. (2009)
