# Supplementary material for: Haplotype Analysis of the First A4V-SOD1 Spanish Family: Two Separate Founders or a Single Common Founder?
Source: Front Genet. 2019 Nov 8;10:1109. doi: 10.3389/fgene.2019.01109 (PMC6857184; doi:10.3389/fgene.2019.01109)
Supplement: Supplementary file 5 [file Table_3.pdf]

Supplementary Table S3. Primer sequences and experimental conditions of the variants genotyped for haplotype analysis

Variant	Analysis Method	Base Change	Product size (bp)	Forward primer 1 (5'>3')	Forward primer 2 (5'>3')	Reverse primer 1 (5'>3')	T annealing (ºC)
rs121912442	Sanger	C/T	367	AGTCATTCCCGGCCACTC		ACCCGCTCCTAGCAAAGGT	62
rs4817415	as-PCR[(2)]	C/A	179	CATCAACAGCCAGCACCTAC	CATCAACAGCCAGCACCTAA	GTGGCTCAGAATGTCAAGCA	60
rs2070422	Sanger	C/T	220	CAGGCTGGTTTTATCCCAAA		CCCATATCTAGCCCTCCAAA	55
rs9974610	as-PCR[(3)]	A/G	159	GCCACCGCACCCGGCCAGTA	GCCACCGCACCCGGCCAGTG	ACCAGCCATGGTCATTCATT	60
rs1041740	as-PCR	C/T	417	GAGAGCCTGTCTACTTGATG	GAGAGCCTGTCTACTTGATA	CGCGGTTTCTAAAGATCCAG	55
rs2833481	as-PCR	T/C	233	GTCATGCCCATGCATTATGC	GTCATGCCCATGCATTATGT	TCGACCTCCCATAGAGTTGG	55
rs2833483	Sanger[(1)]	T/C	315	AATGTCTCCGTTCTTCATTATTCTG		TCGTTGGAAAGATGTGCTTG	55
rs2070424	as-PCR	A/G	242	TAGCTTTGTTAGCTATGACA	TAGCTTTGTTAGCTATGACG	AGGGCTGATGCCACTAAACA	55
rs2833475	Sanger	A/G	249	TGTGGCGCTTAAAGCAAAAT		TTGGAGTGGGAAGACAGCTT	55
rs16988427	as-PCR	T/C	221	GAACCGGTCTCCTTTCCGCT	GAACCGGTCTCCTTTCCGCC	TGGATTGTTGAAGGACCACA	55
rs2070423	as-PCR	C/T	215	CTCCCTTGGTGCCCGAGACG	CTCCCTTGGTGCCCGAGACA	CCAGTTCCTCAGGCTTGACT	55
rs1008270	as-PCR	A/C	162	TGGGACAAATATGGTCTGAT	TGGGACAAATATGGTCTGAG	GTGGGGTTGCCTTTACTCCT	55
rs2173962	as-PCR	A/G	139	TAAGGTGTAACTCTTGCTGA	TAAGGTGTAACTCTTGCTGG	GCAAATGGTAAATGTAAATCCAAA	55
rs4816405	Sanger	C/G	332	TGCTGGCCTCTGTTCTAGGT		ACGAAACTCCACCCACTGTC	55
Internal control	as-PCR		602	AGGCAATGTGCCCACCTA		TCACAATGCACTGAACAGCA	55
CA repeats	Sanger +FA[(4)]	(CA)n	169-171	[6FAM]-GCTCTTAGGTTGCAAATGT TAACCTTGAT		GTTGGATCCCAGTGTTACACGTTG TACT	58
rs202445	Sanger[(4)]	C/T	246	GCTGGGATTATAGGGTTGAA		GGGCTGCTGTTTTAAAAGTC	58

Each 10 µl PCR reaction contained 40 ng genomic DNA, 0.5µM of both forward and reverse primers, and 0.2mM dNTP mix. Cycling conditions consisted of initial denaturing at 95°C for 3 min, followed by a touchdown step with annealing temperatures ranging from 60°C to 55°C, and finally 20 cycles of 95°C for 30 sec., 55°C for 30 sec., and 72°C for 30 sec., followed by a final extension step at 72°C for 5 min. All PCR products were evaluated in 2% agarose gels. PCR products were sequenced in both forward and reverse directions in order to confirm the identified mutations. The primers used for sequencing were the same as those used for PCR amplification. [(1)] PCR primer concentration: 0.25µM; [(2)] touchdown from 62ºC to 60ºC; [(3)] touchdown from 70ºC to 60ºC; (4) Standard PCR (with no touchdown step); as-PCR: Allele specific PCR; FA: Fragment Size Analysis
